# Computer-Assisted Transcatheter Mitral Valve Implantation for Valve-in-Valve Procedures

**DOI:** 10.21470/1678-9741-2023-0237

**Published:** 2024-05-13

**Authors:** Calixte de La Bourdonnaye, Miguel Castro, Clément Joly, Pascal Haigron, Jean-Philippe Verhoye, Amedeo Anselmi

**Affiliations:** 1 LTSI-UMR 1099 - INSERM, University Hospital Center of Rennes - Pontchaillou, Rennes, France

**Keywords:** Mitral Valve Disease, Bioprosthesis, Reoperation, Left Ventricular Outflow Obstruction

## Abstract

Transcatheter mitral valve-in-valve is an alternative to high-risk reoperation on a failing bioprosthesis. It entails specific challenges such as left ventricular outflow tract obstruction. We propose a patient-specific augmented imaging based on preoperative planning to assist the procedure.

Valve-in-valve simulation was performed to represent the optimal level of implantation and the neo-left ventricular outflow tract. These data were combined with intraoperative images through a real-time 3D/2D registration tool. All data were collected retrospectively on one case (pre and per-procedure imaging). We present for the first time an intraoperative guidance tool in transcatheter mitral valve-in-valve procedure.

## INTRODUCTION

**Table:** 

Abbreviations, Acronyms & Symbols	
BVS	= Biological valve stent
CT	= Computed tomography
ES-3	= Edwards SAPIEN 3
LV	= Left ventricular
LVOT	= Left ventricular outflow tract
LVOTO	= Left ventricular outflow tract obstruction
TAVI	= Transcatheter aortic valve implantation
THV	= Transcatheter heart valve
TMVI	= Transcatheter mitral valve implantation
ViV	= Valve-in-valve

Transcatheter mitral valve-in-valve (ViV) is an option for patients at high/prohibitive surgical risk for reoperation on a failing mitral bioprosthesis. As a particular situation of transcatheter aortic valve implantation (TAVI), the ViV procedure has been standardized for the aortic position with satisfactory hemodynamics results.

The implantation of a balloon-expandable transcatheter valve in a mitral bioprosthesis (transcatheter mitral valve implantation [TMVI]) has been proposed. Nonetheless, the anatomical continuity of the mitral valve with the left ventricular (LV) cavity and LV outflow tract (LVOT) imposes specific constraints^[1]^. LVOT obstruction (LVOTO) is a serious^[2]^ complication of any TMVI procedure and depends on patient specific anatomical factors.

Deployment of the transcatheter heart valve (THV) at a suboptimal level inside the bioprosthesis may occur due to limited fluoroscopy markers of failing valves^[2]^, leading to perivalvular leakage, valve migration, or increased LVOTO risk. Overall procedural success at 30 days is only 76.4%^[3]^.

We present a novel proof-of-concept based on a planning workflow and guidance tool aimed at evaluating the LVOTO risk and at providing augmented imaging in TMVI-ViV for improved intraoperative guidance.

## METHODS

### General Information

We collected data from a patient who had received a Carpentier-Edwards (Edwards Lifesciences Corporation, Irvine, California, United States of America) (33 mm) bioprosthesis. After 11 years, he received an Edwards SAPIEN 3 (ES-3) (Edwards Lifesciences Corporation, Irvine, California, United States of America) (nº 29) THV during TMVI-ViV procedure due to structural valve deterioration.

All the data presented below are collected from one case, planning and experience have been done retrospectively on pre and per-procedural imaging.

The patient has given his informed consent for the retrospective use of his clinical data for statistical and research purposes.

### Planning Phase

The first step is to perform a semiautomatic segmentation from preoperative contrast-enhanced computed tomography (CT)-scan to obtain a 3D representation of the aortic root, the left ventricle, and the degenerated biological valve stent (BVS) during systole (region growing algorithm). We applied a segmentation method called region growing^[4]^ to extract the contours and then represented it as surface meshes.

**Fig. 1:**
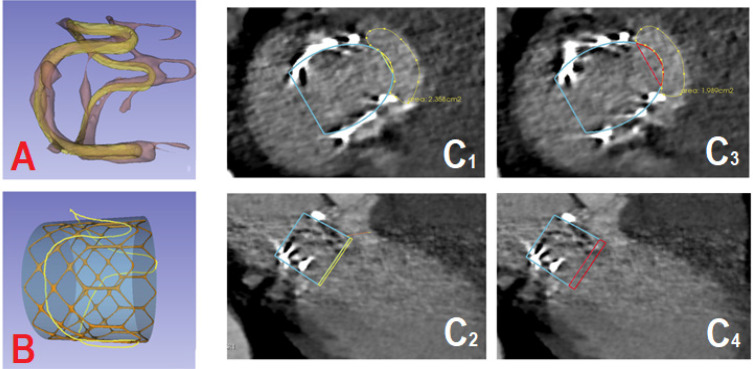
Matching between the degenerate biologic valve stent (BVS)-mesh and one undeteriorated model, simulation of the transcatheter heart valve place on the computed tomography (CT)-scan. (A) The matching between the degenerate BVS-mesh and one undeteriorated BVS-mesh from our library. (B) Simulation of the deployment of an Edwards SAPIEN 3 (ES-3) (Edwards Lifesciences Corporation, Irvine, California, United States of America) valve in the mesh surfacing of the deteriorated BVS. (C1, 2, 3, 4) Simulation of the ES-3 place on the CT-scan with calculation of the neo-left ventricular outflow tract area at each level.

Second, we match the degenerate BVS-mesh and its correspondent undeteriorated BVS-mesh from our library of bioprosthesis^[5]^ (Figure 1A). We applied an iterative closest point algorithm to align the two BVS-meshes. It permits to complete the mesh surfacing of the deteriorated bioprosthesis. Then, we simulate the deployment of an ES-3 valve in the mesh surfacing of the deteriorated BVS (Figure 1B).

The third step was to represent the position of the ES-3 valve on the CT-scan to evaluate its place in the LVOT. The shape and area of the anticipated neo-LVOT are examined (Figure 1C), we considered that the smallest neo-LVOT area should be > 50% of the initial area or > 250 mm^2[2]^.

The level of implantation inside the failing bioprosthesis is simulated.

**Fig. 2:**
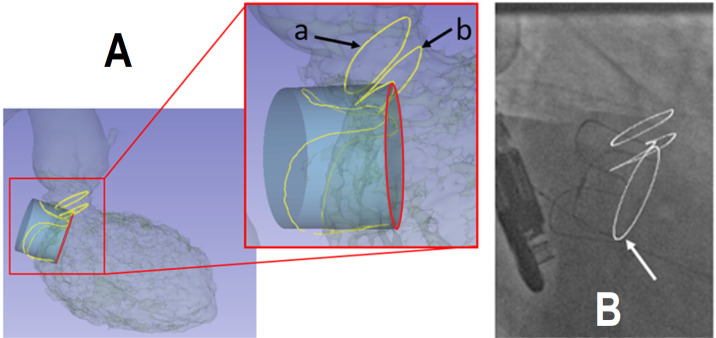
Representation of the left ventricular outflow tract (LVOT) and the ideal implantation level and the augmented fluoroscopic image with three benchmarks. (A) The first diameter is the LVOT ring which still has an ellipsoidal shape and is not obscured by the prosthesis (a). The second is located at the interface between the prosthesis and the LVOT, on the section most obscured by the new prosthesis (b). The blue cylinder represents the device shape and the red circle represent the optimal implantation level (landing zone). (B) The overlaid target plan of implantation (white arrow) and the two LVOT-rings on fluoroscopic image by 2D/3D registration.

We have chosen three benchmarks for the computer phase. Two diameters of the LVOT are delineated. The first is the deepest LVOT diameter which is not obscured by the prosthesis. The second is the smallest LVOT section obscured by the new device, perpendicular to the centerline of the LVOT. The ideal implantation level is also represented (Figure 2A).

## RESULTS

During the simulation, the optimal implantation height is chosen on the preoperative CT-scan. It conciliates stability of the THV and the largest LVOT area. This level and the corresponding LVOT diameters are recorded.

Imported 3D data from the previous steps are combined with the 2D intraoperative fluoroscopic images (Discovery™, General Electric, Boston, Massachusetts, United States of America).

In this retrospective study, we use recorded live fluoroscopic sequences. All surface meshes are imported, including the BVS and the two LVOT contours.

The BVS are observable both in 3D CT and 2D fluoroscopic images.

To perform the registration step, we make a manual selection of three points over the peaks of the BVS on the fluoroscopic image (the information of these points on the 3D-mesh had been acquired in the planning phase).

A registration process was used to determine the rigid 3D/2D transformation by minimizing the distance between the corresponding 3D and 2D points.

The 3D/2D transformation was used to project the preoperative 3D-mesh model and additional landmarks (LVOT contours, implantation level) onto fluoroscopic images.

This was performed through a stand-alone, homemade software^[6]^.

A robust tracking-learning-detection approach with tracking by adaptive appearance model^[7]^ was used to maintain the superimposition on the moving fluoroscopic image.

The result is an augmented reality navigation interface using a 3D-2D registration process during TMVI-ViV procedure. It adds anatomical information such as the implantation level and the LVOT markers to guide the surgeon (Figure 2B).

## DISCUSSION

We propose a computer-assisted tool for improved intraoperative guidance in TMVI-ViV. During the planning phase, we do not only assess the feasibility of the procedure. We have also chosen the ideal level of implantation to improve the neo-LVOT area and the level of implantation for better and predictable hemodynamic outcomes.

Unsatisfying results of the deployed THV may occur during ViV procedures (TAVI-ViV: 10-15% malposition^[8]^, TMVI-ViV: 5% LVOTO^[2]^, 16.3% device unsuccess^[3]^). For TMVI-ViV, a balloon-expandable THV not originally designed for employment in the mitral position is employed. Adding anatomical benchmarks would improve these results on a beating heart.

The 3D/2D fusion of CT-scan derived images onto fluoroscopic images have been used in the context of TAVI-ViV^[5]^. Superimposing a representation of the LVOT during the procedure on live fluoroscopy images (augmented imaging) might help visualizing its relationship with the balloon-expandable THV and guide valve deployment. There is no dedicated tool to evaluate the predicted neo-LVOT after TMVI-ViV, and semiquantitative approaches have considerable error margin in predicting anatomical results^[9]^. With real-time representation of anatomical landmarks on fluoroscopy images, intraoperative adaptations of the deployment level are possible. Adding a representation of the optimal THV deployment level would also optimize the actual level for optimal stability and sealing. The sealing zone of the THV should be deployed at the base of the BVS. Finally, the 3D projection on a 2D image of the diameter of the LVOT enables the selection of fluoroscopy angles. Associating the orthogonal view to the axis of the failing bioprosthesis and the orthogonal view to the centerline of the neo-LVOT would be optimal.

### Limitations

There are limits to our work. First, we projected on fluoroscopy images a representation of the native LVOT rather than of the expected neo-LVOT. Nevertheless, this is expected to help figuring out in real time the neo-LVOT during THV deployment. A second problem is the lack of a view orthogonal to the axis of the deteriorated BVS. This proof-of-concept work needs testing in clinical environment. Several questions are raised; for each patient, highly specialized, costly, and time-consuming evaluation is required^[10]^. Herein, we present a collaborative work involving surgeons and engineers. Other teams had reproduced the LVOT anatomy by a 3D printer or modeling the flow in the LVOT^[10]^, which illustrates the complexity of the planning required^[9]^. To standardize TMVI, patient-specific and semiautomated guiding is needed to improve clinical results, save time, and ameliorate the cost/effectiveness ratio. The current work is part of this process.

## CONCLUSION

Herein we validate the feasibility of a 3D/2D registration tool during the TMVI-ViV procedure. It would allow the surgeon to adapt his/her procedure to live anatomy.

Based on the current work, we plan prospective investigations to verify the feasibility of intraoperative guidance in actual TMVI-ViV.
